# From Code to Life: The AI‐Driven Revolution in Genome Editing

**DOI:** 10.1002/advs.202417029

**Published:** 2025-06-19

**Authors:** Zhidong Li, Wasi Ullah Khan, Genxiang Bai, Chao Dong, Jungang Wang, Youpeng Zhang, Chong Wang, Hongbin Zhang, Wenyi Wang, Ming Luo, Fei Chen

**Affiliations:** ^1^ National Key Laboratory for Tropical Crop Breeding College of breeding and multiplication (Sanya Institute of Breeding and Multiplication) Hainan University Sanya 572025 China; ^2^ College of Tropical Agriculture and Forestry Hainan University Danzhou 571737 China; ^3^ South China Botanical Garden Chinese Academy of Sciences Guangzhou 510650 China; ^4^ School of Life Sciences East China Normal University Shanghai 200241 China; ^5^ National Key Laboratory for Tropical Crop Breeding Institute of Tropical Bioscience and Biotechnology Chinese Academy of Tropical Agricultural Sciences Haikou 571101 China; ^6^ College of Agriculture South China Agricultural University Guangzhou 510642 China

**Keywords:** artificial intelligence, CRISPR, deep learning, genome editing, gene regulation

## Abstract

Genome editing has revolutionized modern biotechnology, enabling precise modifications to DNA sequences with far‐reaching applications in medicine, agriculture, and synthetic biology. Recent advancements in artificial intelligence (AI) have significantly enhanced genome editing by improving target selection, minimizing off‐target effects, and optimizing CRISPR‐associated systems. AI‐driven models, such as deep learning‐based predictors and protein language models, enable more accurate sgRNA design, novel Cas protein discovery, and enhanced gene regulatory network analysis. Additionally, AI‐powered tools facilitate large‐scale data integration, accelerating functional genomics and therapeutic genome editing. This review explores the intersection of AI and genome editing, highlighting key innovations, challenges, and future prospects. Despite its transformative potential, AI‐driven genome editing raises ethical concerns regarding data bias, algorithmic transparency, and unintended genetic modifications. Addressing these challenges requires interdisciplinary collaboration between AI researchers, molecular biologists, and policymakers. As AI continues to evolve, its integration with genome editing will pave the way for groundbreaking advancements in precision medicine, genetic disease treatment, and sustainable agriculture.

## Graphical abstract

This review explores AI‐driven advancements in genome editing, focusing on CRISPR optimization, enhanced targeting precision, reduced off‐target effects, and novel tool design. It addresses ethical challenges, data biases, and highlights future prospects in precision medicine, agriculture, and synthetic biology.

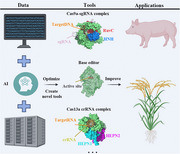

## 1 History and Evolution of Artificial Intelligence

**Figure 1:**
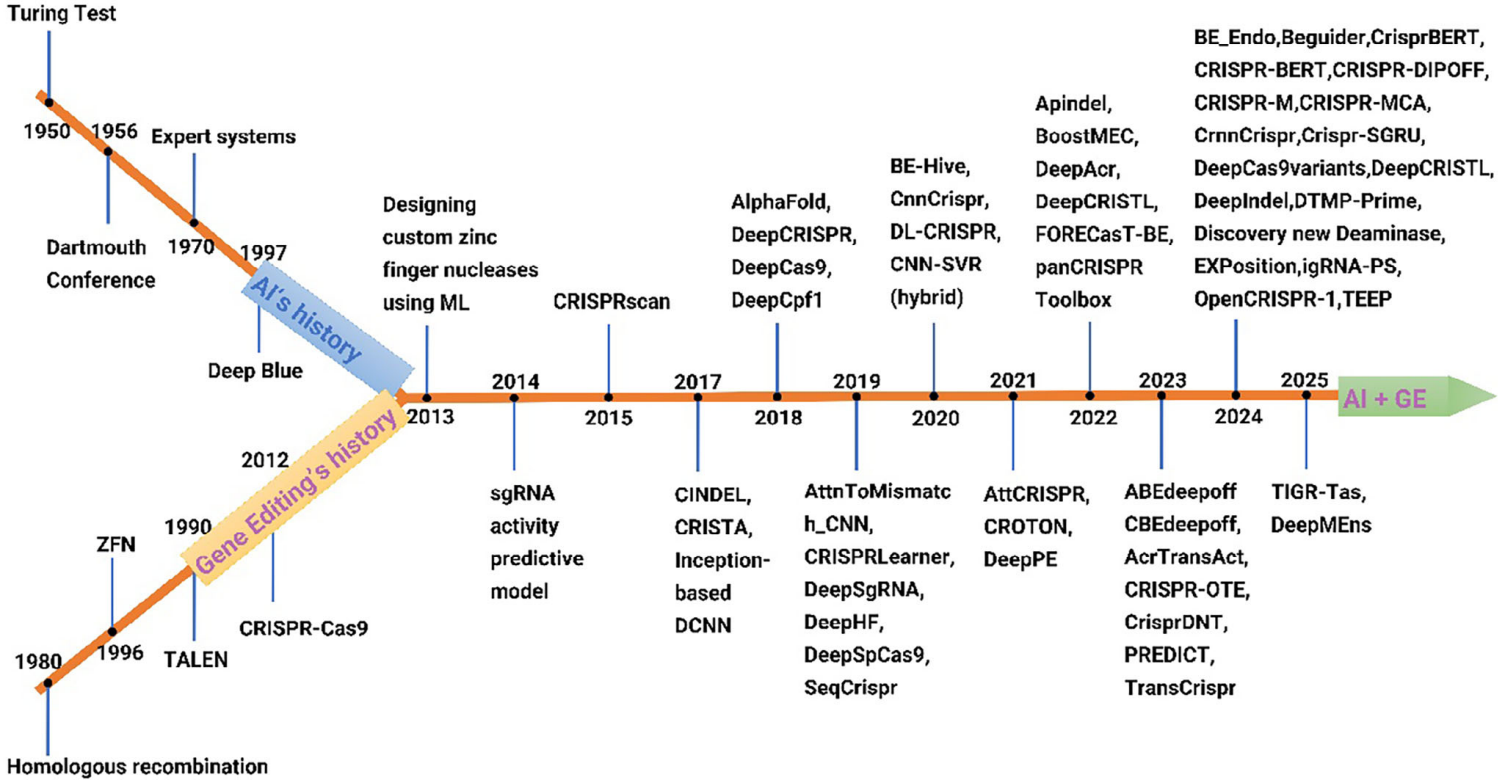
A schematic timeline illustrating the historical evolution and integration of key events in artificial intelligence (AI) and genome editing (GE). Starting with shared milestones highlighted by the orange line and blue markers, the timeline splits into two branches: the yellow “Genome Editing History” branch (annotated in purple) extending to the left, which documents crucial advancements such as CRISPR‐Cas9; and the light blue “AI History” branch (annotated in dark blue) extending to the right, marking breakthroughs like neural networks. The two branches converge around 2013 into a single line, culminating in a green “AI + GE” arrow. Blue markers in the integration zone emphasize future milestones, such as AI‐enhanced editing, against a black background.

The concept of artificial intelligence (AI) predates the emergence of genome‐editing technologies in the life sciences. In 1950, Alan Turing introduced the famous “Turing Test” in his paper to explore whether machines could exhibit intelligence equivalent to that of humans, laying the philosophical groundwork for AI research^[^ ^1^ ^]^ (**Figure** **1**). In 1956, during the Dartmouth Conference, scientists including John McCarthy formally introduced the concept of “Artificial Intelligence (AI).”^[^ ^2^ ^]^ In 2012, Hinton and his colleagues achieved unprecedented results in the ImageNet image recognition competition using deep convolutional neural networks (CNN), sparking a deep learning revolution.^[^ ^3^ ^]^ In 2017, Google introduced the Transformer model, which soon achieved outstanding results in machine translation, text generation, and reading comprehension tasks.^[^ ^4^ ^]^ Transformer‐based models such as BERT and GPT have since excelled in natural language processing (NLP). With the rise of these large models, AI has made groundbreaking progress in dialogue systems, code generation, and text summarization, while also sparking discussions on explainability, bias, and ethics. In the future, as computational power and algorithms continue to advance, AI is poised to further expand its application boundaries, playing an increasingly significant role in fields such as genome editing, drug development, smart manufacturing, and autonomous driving.^[^ ^5^, ^6^ ^]^

## 2 Origin and Evolution of Genome Editing

In the early stages of genome editing, researchers primarily relied on homologous recombination for gene targeting.^[^ ^7^ ^]^ Although this process is widespread in nature, its efficiency is generally low, making it difficult to apply to more complex mammalian cells.^[^ ^8^ ^]^ In the early 1980s, scientists gradually developed methods to perform gene knockouts or modifications in the laboratory using homologous recombination, thereby laying the foundation for later targeted gene editing technologies (Figure 1). Zinc Finger Nucleases (ZFNs), the first generation of artificial site‐specific nucleases, began to emerge in the 1990s.^[^ ^9^ ^]^ The advent of ZFNs marked the first time that researchers could “cut” the genome at specific sites by design in the laboratory, greatly enhancing the precision of gene modifications. However, the design and assembly of ZFNs were relatively complex, and their off‐target effects were quite pronounced, which limited their broader application. To overcome the challenges of design complexity and off‐target effects associated with ZFNs, scientists developed Transcription Activator‐Like Effector Nucleases (TALENs). Similar to ZFNs, TALENs also use the FokI enzyme to complete DNA cleavage. TALENs are simpler to design than ZFNs and exhibit greater versatility in recognizing diverse DNA sequences.^[^ ^10^ ^]^ They have been successfully applied in mammalian cells and model organisms (such as zebrafish and mice), opening up new possibilities for gene editing in both basic research and potential clinical treatments.^[^ ^11^ ^]^

In 2012, Jennifer Doudna, Emmanuelle Charpentier, and their colleagues elucidated the crucial role of the CRISPR‐Cas9 system in bacterial defense against phage invasion, proposing that it could be repurposed as a universal gene editing tool.^[^ ^12^ ^]^ In 2013, Feng Zhang's team was the first to demonstrate the effectiveness of CRISPR‐Cas9 in eukaryotic cells (human cells), ushering in a new era of gene editing.^[^ ^13^ ^]^ CRISPR‐Cas9 offers the advantages of simple design, high efficiency, low cost, and strong scalability, significantly lowering the barrier to genome editing.^[^ ^14^ ^]^ Researchers need only design a single guide RNA (sgRNA) corresponding to the target DNA sequence to “direct” the Cas9 enzyme to a specific gene locus for cleavage. This revolutionary technology has generated tremendous enthusiasm across agriculture, medicine, and fundamental biology.^[^ ^15^ ^]^

**Figure 2:**
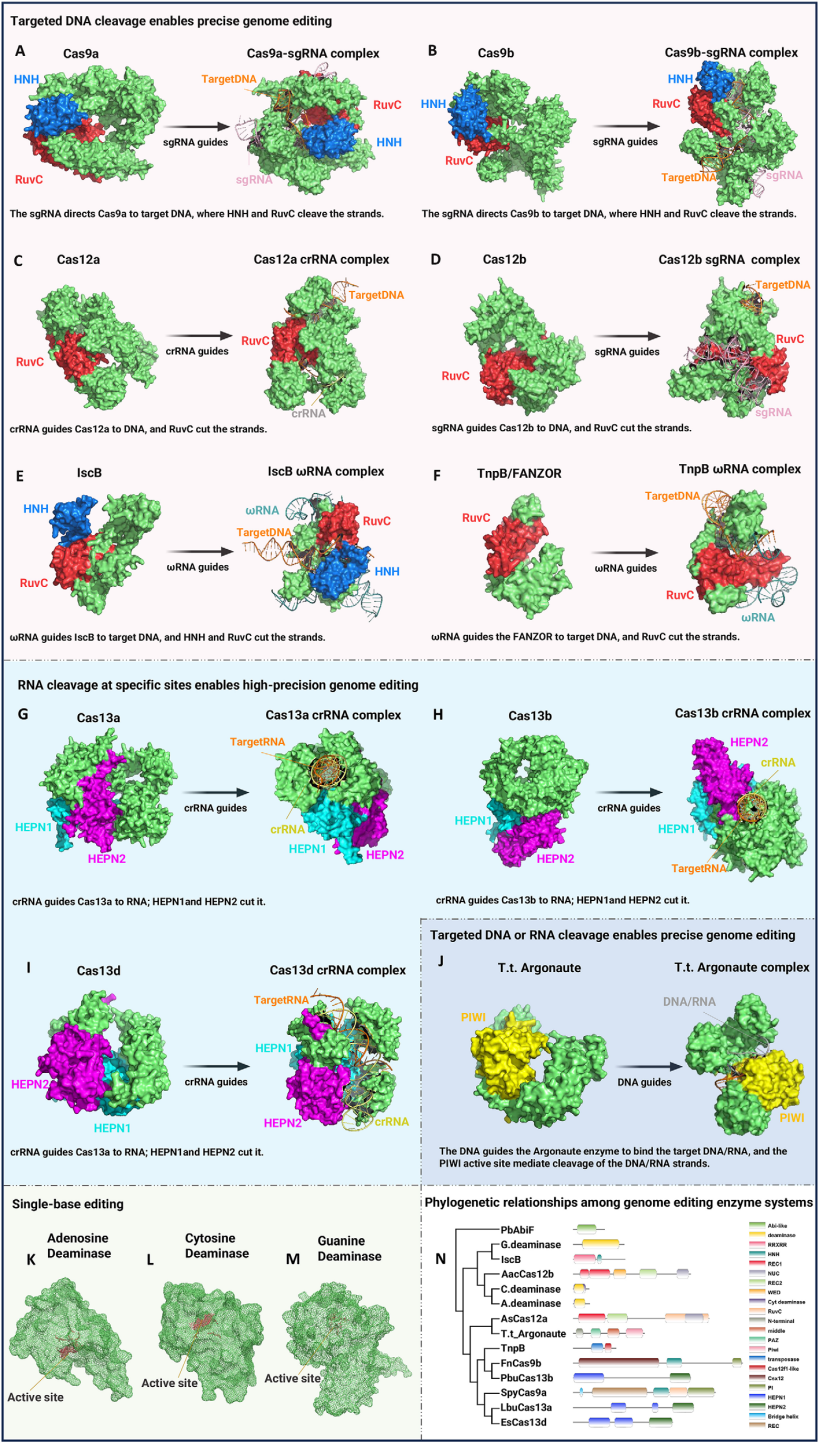
Gene editing toolbox and operating principles. A–F) DNA‐targeting systems, this section illustrates the structures of various CRISPR systems, related components, and simplified schematic representations. Each box depicts the corresponding Cas proteins (including Cas9a (PDB: 8KAM),^[^ ^29^ ^]^ Cas9b (PDB: 5B2O),^[^ ^30^ ^]^ Cas12a (PDB: 8SFP),^[^ ^31^ ^]^ Cas12b (PDB: 5U30),^[^ ^32^ ^]^ IscB (PDB: 8CSZ),^[^ ^33^ ^]^ TnpB (PDB: 8H1J)^[^ ^34^ ^]^) and the complexes they form with guide RNAs (e.g., sgRNA, crRNA). Color coding is used to highlight the functional domains. G–I) RNA‐targeting systems, crRNA directs Cas13 (Cas13a (PDB: 5XWP^[^ ^35^ ^]^), Cas13b, Cas13d (PDB: 6E9F^[^ ^36^ ^]^)) enzymes to RNA; HEPN1 and HEPN2 domains mediate cleavage. J) Argonaute complex (PDB: 3HVR)^[^ ^37^ ^]^ is a DNA‐guided nuclease system, cleave the target DNA or RNA strands. K–M) Single‐base editing, deaminases catalyze base conversion at active sites for precise genetic modification. N) This panel presents the phylogenetic tree of the aforementioned proteins and their functional domains, including four characteristic domains: N, PAZ, MID, and PIWI.

With the widespread application of CRISPR‐Cas9 across various biological systems, researchers have continuously delved into and modified different types of Cas proteins, aiming for higher editing efficiency and specificity in diverse contexts. As depicted in the **Figure** **2**, the 3D structures of Cas9, Cas12 (Cpf1), Cas13, and other novel Cas family enzymes each exhibit unique features (as shown in Figure 2A–N). They differ in target recognition and cleavage mechanisms: Cas9 typically targets DNA.^[^ ^12^, ^16^ ^]^ Cas12a produces 5’ overhangs when cleaving the complementary strand,^[^ ^17^ ^]^ and Cas13 specializes in recognizing and cleaving RNA.^[^ ^18^ ^]^ This diversity allows researchers to choose the most suitable enzyme tool for operations at the DNA or RNA level (Figure 2O–AH) based on experimental needs. Moreover, to avoid reliance on double‐strand breaks, inactivated or partially inactivated versions of Cas9 (dCas9) have been widely applied in CRISPR interference (CRISPRi)^[^ ^19^ ^]^ and CRISPR activation (CRISPRa).^[^ ^20^ ^]^ By fusing dCas9^[^ ^21^ ^]^ with transcriptional repression or activation domains (represented in the figure by different colored functional domains, such as adenosine deaminase and cytochrome proteins), researchers can finely tune target gene expression levels without permanently altering the gene itself.^[^ ^22^, ^23^ ^]^ At the same time, the rise of CRISPR screening technologies has greatly expanded the dimensions of gene function research and drug target discovery.^[^ ^24^ ^]^ By combining CRISPR with large‐scale libraries, thousands of gene loci can be simultaneously knocked out or modulated in a short period, with key genes related to specific phenotypes rapidly identified through high‐throughput sequencing.

In the ongoing pursuit of higher precision and lower off‐target rates, gene editing technology has witnessed a revolutionary wave with the advent of Base Editing^[^ ^25^ ^]^ and Prime Editing.^[^ ^26^ ^]^ As shown in Figure 2, the key enzymes used in base editing—adenosine deaminase and cytosine deaminase—are typically fused with inactivated or partially inactivated Cas9, enabling precise single‐base substitutions (A→G or C→T).^[^ ^27^ ^]^ In 2016, David Liu's team first reported this method, ingeniously using dCas9 or Cas9 nickase to locate the target base and precisely guide the deaminase to the editing region, significantly reducing the cytotoxicity and off‐target risks associated with double‐strand breaks.^[^ ^28^ ^]^ Then in 2019, the same team introduced Prime Editing by fusing reverse transcriptase to a Cas9 nickase and using a “prime editing guide RNA” that carries the desired modification template, allowing for precise insertions, deletions, and substitutions within the genome. Compared to traditional CRISPR‐Cas9, these novel editing tools have not only achieved significant breakthroughs in precision and safety but also provided more flexible application strategies for various fields, including clinical gene therapy, plant and animal breeding, and functional genomics. As illustrated by the composite structures of different Cas enzymes, RNAs, and deaminases in the figure, the evolution of CRISPR technology has entered a new era of refinement and multifunctionality, laying a solid foundation for further integration with AI algorithms and the development of higher throughput, lower‐cost personalized gene editing solutions.^[^ ^5^ ^]^

## 3 The Convergence and Applications of AI and Genome Editing

### 3.1 The Convergence of AI and Genome Editing

The deep integration of artificial intelligence (AI) and genome editing originally stemmed from the dual demands for high throughput and high precision. After the successful application of CRISPR‐Cas9 in mammalian cells between 2012 and 2013, many laboratories began accumulating large‐scale data on off‐target effects and target screening. However, the complexity of this data often exceeded the processing capabilities of traditional statistical methods. At that time, deep learning made breakthrough advances in image recognition in 2012, and the proliferation of GPUs and cloud computing provided unprecedented computational power for training and analyzing massive biological datasets. As a result, the research community gradually recognized that AI algorithms could be leveraged to identify key patterns and variables hidden within the gene editing process.

In 2013, the development of the sgRNA predictive model—likely involving early AI techniques—served as a foundational step for CRISPR‐Cas9 optimization, while Doench et al.’s landmark 2014 paper explicitly employed machine learning to enhance sgRNA design, marking a significant fusion of AI and genome editing.^[^ ^38^ ^]^ In 2015, CRISPRscan utilized randomized logistic regression for feature selection and applied a linear regression model to predict sgRNA activity.^[^ ^39^ ^]^

Following AlphaGo's victory over top professional players in 2016, the potential of AI gained even greater attention from both the public and academia, spurring widespread adoption of deep learning and reinforcement learning techniques. In the field of genome editing, this technological transfer has manifested in the automated analysis of large‐scale CRISPR screening data: researchers now center their work around machine learning to integrate transcriptomic, proteomic, and epigenomic information, construct predictive models, and assess the performance and risks of gene editing across diverse cellular environments. At the same time, synthetic biology in the latter half of the 2010s began harnessing AI for protein structure prediction and metabolic pathway simulation, providing novel strategies for customized enzyme engineering and cell factory development.

Today, with more refined editing tools such as Prime Editing being introduced, AI's role in safety evaluation and the design of personalized treatment strategies has become increasingly prominent. AI can rapidly match each patient with the optimal potential target and accurately predict possible side effects. For example, the development of DTMP‐Prime reflects the integration of AI and prime editing.^[^ ^40^ ^]^ Clearly, AI is acting as a “navigator,” leading genome editing from basic research into clinical applications, while genome editing, in turn, supplies rich and diverse biological data that further advances AI. Together, they are continually expanding the frontiers of medicine and biotechnology.

### 3.2 General Paradigm of AI in Genome Editing Research

**Figure 3:**
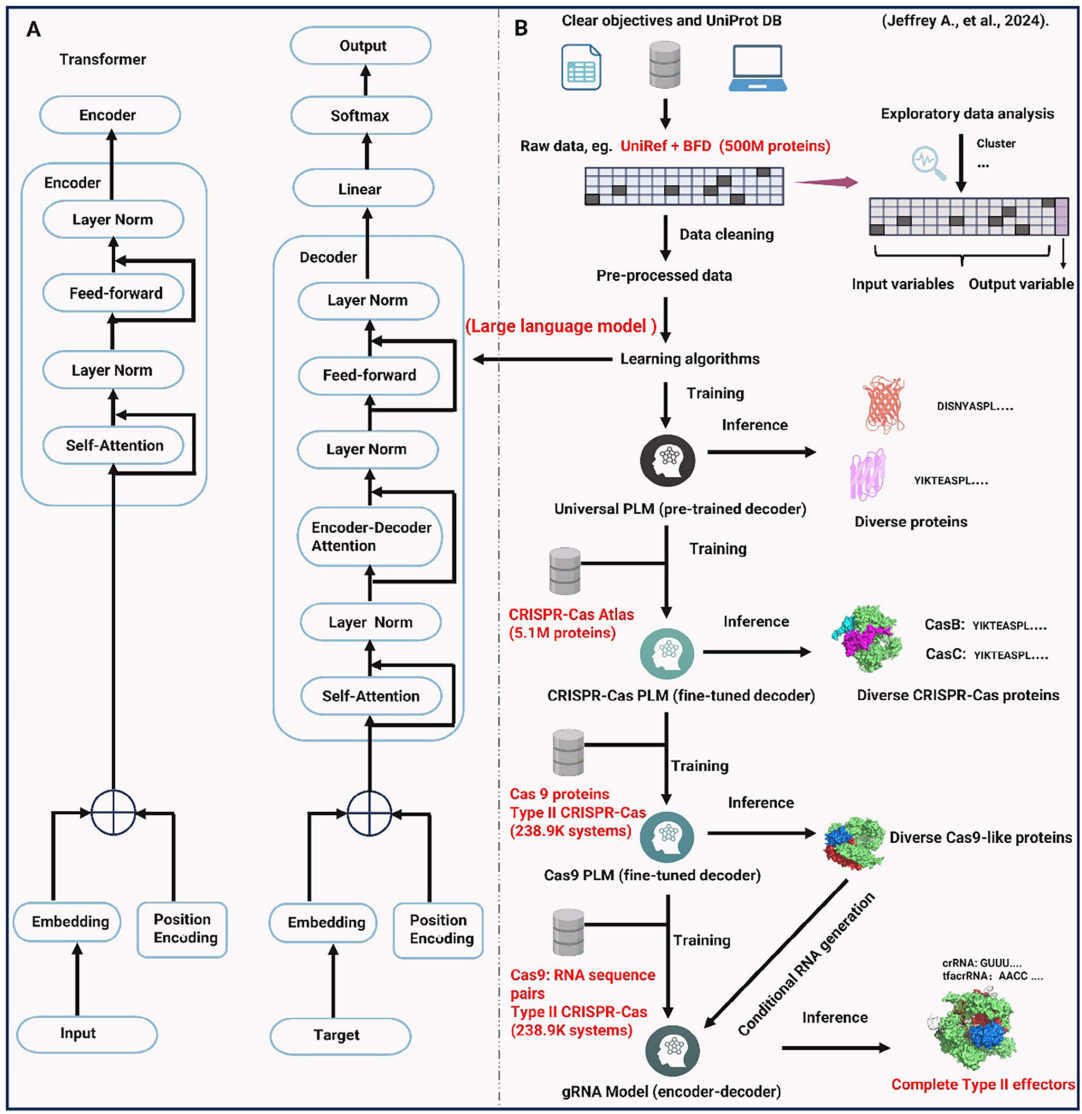
General paradigm of AI integration in genome editing. A) Diagram of a transformer‐based architecture for AI‐driven genome editing. The model employs an encoder‐decoder structure with self‐attention mechanisms, layer normalization (Layer Norm), and feed‐forward networks. B) This workflow illustrates the application of language models (LMs) in designing CRISPR‐Cas systems. The process begins with pre‐training on diverse protein datasets, such as UniRef and BFD (500M proteins), to learn general constraints of protein evolution across the evolutionary tree. The pre‐trained universal protein language model (PLM) is then fine‐tuned on CRISPR‐Cas‐specific datasets, including Cas9 proteins (238.9K systems), Cas9‐like proteins, and Cas9‐gRNA sequence pairs (238.9K systems). Through exploratory data analysis and clustering, the model identifies input and output variables, enabling inference to generate diverse CRISPR‐Cas proteins and gRNA sequences. The fine‐tuning process enhances the model's ability to design complete Type II effectors, improving the precision and diversity of CRISPR‐Cas systems for genome editing applications.^[^ ^49^ ^]^

At the forefront of modern genome editing research, artificial intelligence—particularly deep learning and large‐scale pre‐trained models—is providing unprecedented support for exploring and designing novel CRISPR tools.^[^ ^5^ ^]^ As illustrated in **Figure** **3**, the AI‐powered genome editing research paradigm can be summarized into several closely interconnected stages: dataset construction and preprocessing, large model pre‐training, task‐specific fine‐tuning, and ultimately, inference and application in genome editing scenarios.^[^ ^41^ ^]^ These steps are visually represented in Figure 3A,B: Figure 3A delves into the core structure of the Transformer model,^[^ ^4^ ^]^ highlighting how key mechanisms such as self‐attention, feed‐forward networks, and positional encoding process sequential data. Meanwhile, Figure 3B demonstrates how protein databases (e.g., UniProt) and CRISPR‐related datasets undergo pre‐training and fine‐tuning, ultimately enabling precise design and evaluation of Cas proteins and their guide RNAs.

#### 3.2.1 Dataset Construction and Preprocessing

In the initial stage, researchers collect vast amounts of protein or nucleic acid sequence data from large public databases such as UniProt, PDB, and CRISPR‐Cas Atlas. Since CRISPR systems involve Cas family proteins, sgRNA sequences, and various transcriptional regulatory elements, relying on a single data source is insufficient to fully capture the biological complexity. To enhance AI models’ ability to recognize structural and functional patterns, researchers integrate sequence data with 3D structures, annotation details (e.g., protein functional domains, evolutionary conservation), and experimentally measured activity data. These datasets are then encoded or labeled in a way that aligns with Transformer architectures—such as mapping amino acid or nucleotide sequences into discrete tokens.^[^ ^42^ ^]^ During this process, high‐quality data cleaning and feature engineering play a crucial role in improving model performance.^[^ ^43^ ^]^

#### 3.2.2 Pre‐Training of Large Models

Once the data is prepared, researchers utilize Transformer^[^ ^4^ ^]^ architectures to pre‐train models on large‐scale sequences. The Encoder‐Decoder module shown on the left side of Figure 3A is a common choice, though Encoder‐only or Decoder‐only variants may also be used. The goal of pre‐training is to enable the model to gradually learn the semantic and syntactic rules governing protein or RNA sequences through large‐scale unsupervised learning. Similar to natural language processing, the model leverages self‐attention mechanisms to capture long‐range dependencies within sequences, forming a contextual understanding of amino acids and nucleotides. After completing this stage, the model acquires a generalized representation of sequences, making it transferable to more specialized CRISPR applications. Besides, Kaiyi Jiang et al. proposed the EVOLVEpro framework, which integrates protein language models with activity predictors to rapidly enhance protein activity using only a small amount of experimental data. This method significantly improves editing efficiency by optimizing CRISPR nucleases and Prime Editors.^[^ ^44^ ^]^

#### 3.2.3 Fine‐Tuning for Genome Editing

The next phase is fine‐tuning for genome editing tasks. At this stage, researchers extract generalized sequence encodings from the pre‐trained model and further train it with task‐specific CRISPR data using supervised or semi‐supervised learning. For instance, to predict off‐target risks of Cas9 or Cas12 enzymes, experimental off‐target sites, and corresponding sequences can be input into the model for classification or scoring.^[^ ^24^ ^]^ Similarly, for optimizing sgRNA design, sgRNA sequences can be trained alongside editing efficiency and cytotoxicity indicators, allowing the model to assess the potential performance of each sgRNA.^[^ ^45^ ^]^ The right half of Figure 3B illustrates this approach: first, “universal” sequence representations are obtained from large protein databases (Universal PLM), then fine‐tuned for different CRISPR types or protein variants to generate precise predictions and recommendations.^[^ ^5^ ^]^ During this process, large models can leverage previously learned protein structural information to suggest modifications for Cas protein functional domains or capture RNA secondary structure features to assist in sgRNA sequence optimization.^[^ ^39^, ^46^ ^]^

#### 3.2.4 Inference and Applications

Once trained, the model can be directly applied to various genome editing scenarios. On one hand, it can automatically screen and rank high‐specificity, low off‐target sgRNA sequences for subsequent experimental validation.^[^ ^24^ ^]^ On the other, it can assist researchers in designing and engineering novel Cas proteins or base‐editing enzymes, expanding the toolkit for clinical gene therapy and synthetic biology.^[^ ^5^ ^]^ Given that the Transformer architecture has the potential to model multimodal information,^[^ ^4^ ^]^ researchers can also incorporate molecular dynamics simulations, epigenomic data, and transcriptomic data into the model,^[^ ^47^ ^]^ enabling more accurate predictions of genome editing outcomes across different cellular environments and disease states.

At the same time, as model parameter scales increase and algorithms evolve, researchers are exploring ways to make these pre‐trained models more “creative.” Rather than merely evaluating and screening existing sequences, AI is now being employed in generative tasks to “design” novel Cas proteins or RNA structures. This marks a shift where artificial intelligence is seamlessly integrated into the genome editing tool development pipeline. Under this continuously evolving paradigm, the convergence of AI and genome editing will continue to drive transformative advances in precision medicine, agricultural breeding, and industrial biotechnology.^[^ ^48^ ^]^

### 3.3 AI for Precise Identification of Gene Editing Targets and Optimization of Gene Editing Strategies

**Figure 4:**
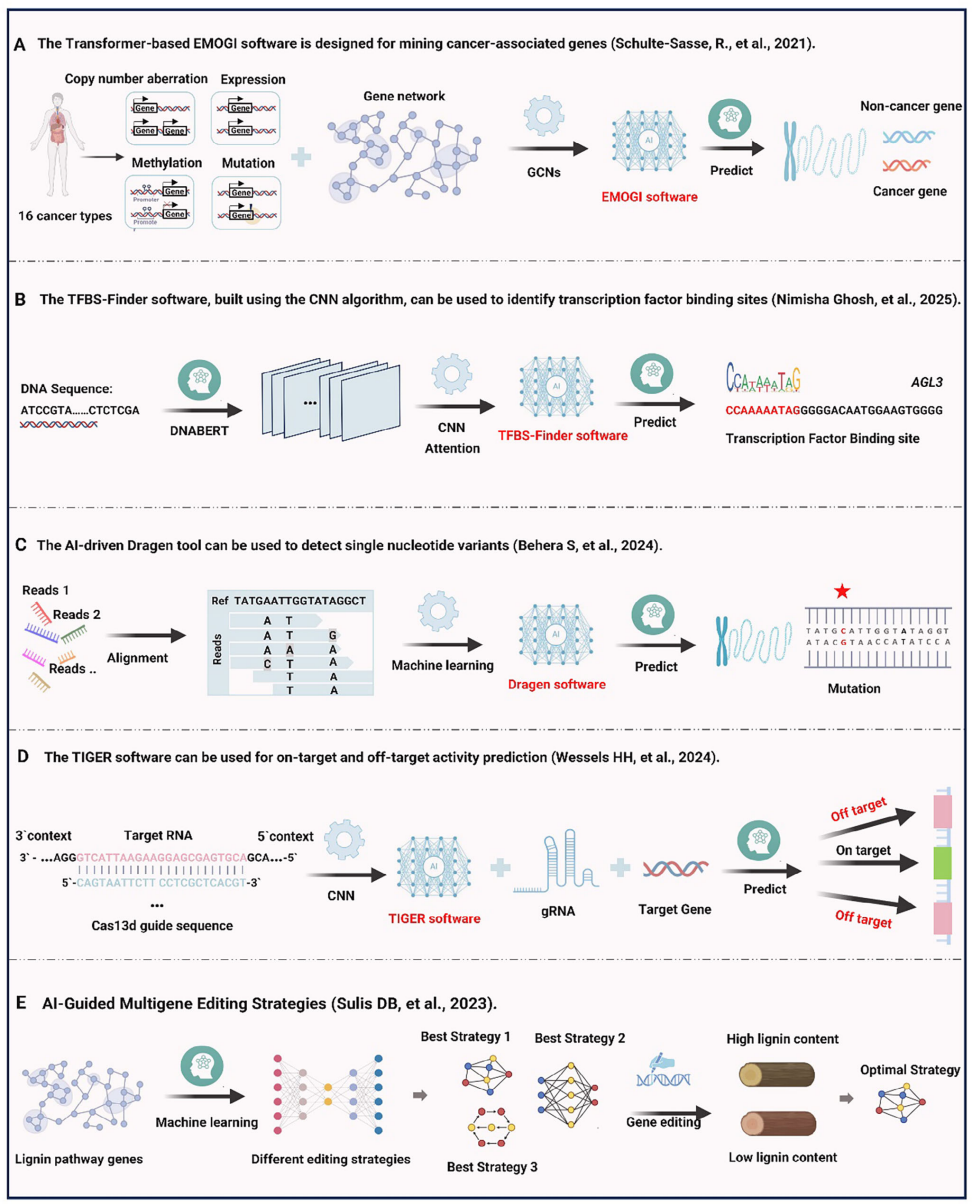
Multifaceted applications of artificial intelligence in gene editing: From gene mining to precision targeting. A) Gene identification. The EMOGI algorithm^[^ ^50^ ^]^ integrates multi‐omics data from patient samples—including somatic mutation profiles, DNA methylation patterns, single‐gene expression signatures, and protein interaction networks across cellular pathways. By employing deep learning, EMOGI uncovers cancer‐driving patterns and molecular mechanisms, enabling the identification of novel cancer‐associated genes through predictive modeling. B) Motif prediction. The DNABERT model^[^ ^51^ ^]^ processes input DNA sequences to capture long‐range dependencies and extract sequence embeddings. Using convolutional neural networks (CNNs) and attention mechanisms, the TFBS‐Finder tool predicts transcription factor binding sites (TFBS). An example is shown for the AGL3 gene, highlighting the model's capability to classify and predict TFBS with precision. C) Variant detection. The Dragen tool^[^ ^52^ ^]^ enhances variant calling by combining sequence alignment‐based identification of genomic loci with machine learning models. These models are trained to detect single‐nucleotide variants (SNVs) with high accuracy, improving the reliability of genomic mutation analysis. D) On‐target and off‐target activity prediction. The TIGER model,^[^ ^53^ ^]^ built on a CNN‐based algorithm (Targeted Inhibition of Gene Expression via Guide RNA design), predicts on‐target efficacy by analyzing guide RNA sequences within their genomic context. Additionally, TIGER evaluates off‐target risks, providing a comprehensive framework for optimizing the safety and efficiency of gene editing applications. E) AI‐guided multigene editing strategies. AI‐enabled combinatorial editing strategies were successfully implemented in Populus to reduce lignin content for sustainable biomass production.^[^ ^54^ ^]^ Machine learning models screened 21 lignin biosynthesis‐related genes to prioritize synergistic editing targets. Researchers selected seven optimal multigene knockout strategies based on predictive scores, with experimental validation confirming edited lines lignin content was reduced.

The core of gene editing technology lies in the targeted modification of specific DNA sequences. Accurately identifying gene editing targets directly determines the effectiveness and safety of the editing process. Efficient analysis of massive, high‐dimensional, unstructured, semi‐structured, and multi‐source heterogeneous data poses a major challenge for the precise identification of editing targets. Traditional research methods, such as multi‐omics analysis, sequence homology comparison, and experimental validation, often struggle to efficiently and accurately identify gene editing targets when faced with vast and complex biological datasets. AI, with its core strengths in processing large‐scale data, recognizing complex patterns, and predicting unknown features, enables the precise identification of gene editing targets. It fundamentally transforms life science research and biotechnology applications, offering revolutionary tools for precision medicine, agricultural improvement, and biomanufacturing. AI has already been widely applied in identifying targets such as genes, mutation sites, and functional domains (**Figure** **4**).^[^ ^50^, ^51^, ^52^, ^53^, ^54^ ^]^

### 3.4 AI for Optimizing Genome Editing Tools

**Figure 5:**
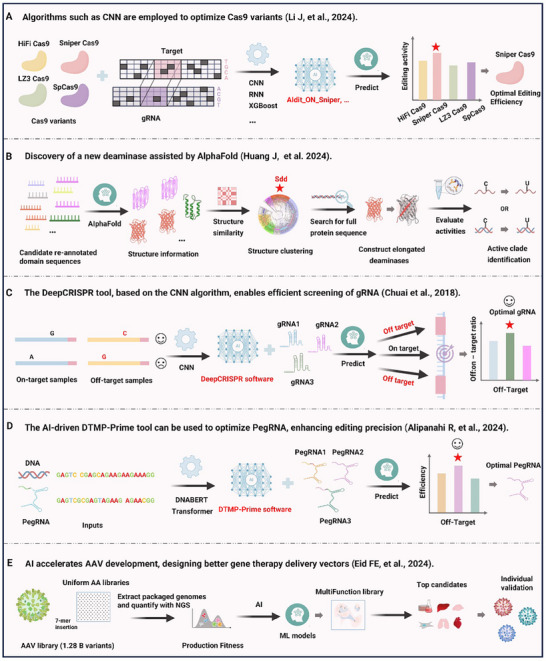
Artificial intelligence‐powered optimization of gene editing tools for enhanced precision and efficiency. A) Optimization of Cas9 variant selection: Machine learning‐guided predictive models streamline the selection of Cas9 variants by enabling both the discovery of novel homologous nucleases across diverse species and the rational engineering of Sniper‐Cas9‐derived mutants through codon redesign. A large‐scale oligo library of in vitro synthesized gRNA‐target pairs was developed, empowering a machine learning‐driven computational framework to enhance Cas9 mutant optimization and selection.^[^ ^55^ ^]^ B) Deaminase engineering and optimization. AI‐driven approaches have revolutionized the development of next‐generation base editors. Using AlphaFold, researchers^[^ ^56^ ^]^ predicted the 3D structures of deaminase functional domains from amino acid sequences, followed by structural similarity‐based clustering. Enzymes were classified into distinct deaminase families based on conserved structural features. Within the SCP1.201 cluster, the Sdd protein demonstrated robust single‐stranded DNA deamination activity, which was experimentally validated to engineer novel programmable base editors. C) gRNA Optimization and Selection: The DeepCRISPR framework^[^ ^57^ ^]^ leverages convolutional neural networks (CNNs) trained on paired on‐target and off‐target activity datasets. This deep learning model simultaneously predicts on‐target efficacy and off‐target risks of candidate gRNAs, enabling the data‐driven selection of high‐precision guide RNAs for CRISPR‐based applications. D) PegRNA design optimization: The DTMP‐Prime tool^[^ ^40^ ^]^ employs a deep Transformer architecture to predict and optimize prime editing guide RNA (pegRNA) designs, significantly enhancing editing efficiency. By processing PegRNA‐DNA sequence pairs through the DNABERT language model, the system captures global statistical patterns of DNA sequences. Following feature selection, the Transformer‐based predictor evaluates prime editing outcomes, offering actionable insights for rational pegRNA engineering. E) This study used the Fit4Function machine learning approach to construct an unbiased AAV capsid library and perform in vitro and in vivo screening to generate high‐quality functional data. ML models were trained to predict multi‐trait variants, selecting AAV capsids with both high manufacturability and liver‐targeting efficiency. These optimized variants were validated in mice and macaques, demonstrating improved cross‐species gene delivery compared to AAV9.^[^ ^58^ ^]^

AI, with its powerful data processing capabilities and predictive accuracy, is revolutionizing the optimization of genome editing tools. In particular, AI has significantly enhanced the precision, efficiency, and specificity of CRISPR‐based technologies (**Figure** **5**).

#### 3.4.1 AI‐Guided Cas9 Variant Selection

AI has demonstrated remarkable capabilities in optimizing key CRISPR components, particularly Cas9 variants. For example, Li et al.^[^ ^55^ ^]^ leveraged convolutional neural networks (CNNs) and recurrent neural networks (RNNs) to systematically evaluate Cas9 variants, including HiFi Cas9, Sniper Cas9, LZ3 Cas9, and SpCas9. By inputting target DNA sequences and guide RNAs (gRNAs) into an AI model and employing algorithms such as Adit_ON_SNIPER and XGBoost, the researchers accurately predicted editing outcomes. Their findings revealed that Sniper Cas9 emerged as the most efficient, highlighted by a star‐marked bar in the results, indicating both superior on‐target activity and significantly reduced off‐target effects. This AI‐driven screening approach functions like a “genome editing referee,” precisely selecting the most suitable tool from a pool of candidates, laying the groundwork for high‐fidelity genome editing.

#### 3.4.2 AI‐Powered Discovery of New Editing Enzymes

Beyond optimizing existing tools, AI is pushing the boundaries of genome editing by identifying novel editing components. Huang et al.^[^ ^56^ ^]^ demonstrated a breakthrough application of AlphaFold in this field. By predicting the structural information of candidate deaminases, they discovered a new base‐editing enzyme, Sdd. The workflow started with candidate domain sequences, followed by structural similarity clustering, which identified Sdd as a promising enzyme. After constructing its full‐length protein sequence and validating its activity, the study classified active (C) and inactive (U) variants. This approach is akin to using AI to create a “protein treasure map”, accelerating enzyme discovery while reducing the trial‐and‐error of traditional experiments, ultimately enriching the genome editing toolkit with powerful new molecular tools.

#### 3.4.3 Precision Targeting to Minimize Off‐Target Effects

The success of genome editing hinges on well‐designed gRNAs, an area where AI excels. Chuai et al. developed DeepCRISPR,^[^ ^57^ ^]^ a CNN‐based tool that analyzes on‐target and off‐target samples to predict gRNA performance. Their results, visually presented in bar charts, highlight the top‐performing gRNAs with high target‐to‐off‐target ratios, marked with a smiley face icon. This method effectively addresses one of CRISPR's greatest challenges—off‐target effects—by ensuring gRNAs function like precision‐guided missiles, striking only intended targets without collateral damage.

Further advancing this field, Wessels et al.^[^ ^53^ ^]^ introduced TIGER, which optimizes CRISPR‐Cas13 systems by predicting RNA targeting context effects. Their model distinguishes on‐target (green) and off‐target (red) activities, offering a refined approach for RNA editing applications.

#### 3.4.4 Prime Editing Optimization

AI is also accelerating the development of next‐generation genome editing technologies, such as prime editing. Alipanahi et al.^[^ ^40^ ^]^ introduced DTMP‐Prime, a tool based on the Transformer model DNABERT, designed to optimize prime editing guide RNA (pegRNA) selection. By inputting DNA sequences and pegRNAs, the model predicts editing efficiency, with the best‐performing pegRNAs (marked with stars in bar charts) demonstrating the lowest off‐target rates and highest precision. This advancement further enhances prime editing—a technique that enables precise modifications without inducing double‐stranded DNA breaks—by leveraging AI‐driven Transformer algorithms to tailor solutions for complex editing tasks.

#### 3.4.5 AI‐Driven AAV Vector Engineering

AI‐based methods are revolutionizing the design of adeno‐associated virus (AAV) delivery vectors by enabling systematic and efficient capsid engineering. Traditional approaches to AAV capsid selection are limited by the vast sequence space, making it challenging to identify variants that optimize multiple essential traits such as manufacturability, targeted transduction, and cross‐species functionality. Machine learning (ML)‐driven strategies, like the Fit4Function framework, address this bottleneck by integrating high‐throughput sequencing data with predictive modeling. By leveraging a diverse and unbiased capsid library, AI models can accurately map sequence variations to functional traits, allowing for the rational design of AAV variants with enhanced gene delivery efficiency. These models not only improve the precision of capsid selection but also facilitate cross‐species generalization, reducing the reliance on costly and time‐consuming animal trials. Ultimately, AI‐guided AAV engineering paves the way for more effective and clinically translatable gene therapy vectors.^[^ ^58^ ^]^

Overall, AI, through advanced algorithms such as CNN, RNN, AlphaFold, and Transformer, is integrated into every stage of genome editing—optimizing variants, discovering new enzymes, designing gRNAs, revolutionizing techniques, and enabling multi‐gene regulation. Its innovation lies in transforming vast biological datasets into highly accurate predictions, while its completeness is reflected in the seamless workflow from sequence input to result validation. Moreover, its readability is enhanced by intuitive result visualization and logically structured advancements. The synergy between AI and genome editing is paving new pathways for therapeutic genome editing, agricultural improvements, and synthetic biology, heralding an era of greater precision, efficiency, and innovation in genetic engineering.

### 3.5 AI Models Applied to Genome Editing

With its powerful data analysis and predictive capabilities, AI has significantly enhanced the precision, efficiency, and specificity of gene editing in various cases (**Table** **1**).

**Table 1:** AI‐based tools for Genome editing applications.

Name of the method	Algorithm used	Applications	Limitations	Refs.
Predict on‐ and off‐target efficiency				
CRISPRscan	LR	On‐target efficiency	Optimized for zebrafish, may lack accuracy in other species.	Moreno‐Mateos etal., 2015 ^[^ ^39^ ^]^
CINDEL	LR	On‐target efficiency	May be inaccurate for guide RNAs with extreme GC content.	Kim et al, 2017 ^[^ ^64^ ^]^
DeepCpf1	CNN	On‐target efficiency	Its performance heavily relies on chromatin accessibility data, limiting accuracy in cell lines lacking such information.	Kim et al., 2018 ^[^ ^65^ ^]^
CNN & FNN, Random Forest, GBTs, & LR	CNN, FNN, GBTs, and LR	off‐target	Limited by the quality of the training data, potentially lacking sufficient generalization to unseen sequences, and its predictive accuracy for extremely low‐similarity distant sequences remains uncertain.	Lin et al., 2018 ^[^ ^66^ ^]^
CRISPRLearner	CNN	Predict CRISPR/Cas9 sgRNA On‐Target Cleavage Efficiency	model bias risks might restrict scalability in real‐world applications with heterogeneous datasets.	Giovanni et al., 2019 ^[^ ^67^ ^]^
AttnToMismatch_CNN	Attention‐based Transformer and CNN	Predict sgRNA on and off‐target specificity	May face data dependency, limited generalization, high complexity, off‐target prediction issues, interpretability challenges, bias, and overfitting.s	Liu et al., 2019 ^[^ ^59^ ^]^
SeqCrispr	RNN + CNN + transfer learning	Predict on‐target efficiency	Model's efficiency may be impacted by insufficient understanding of fluctuating gene activity and phenotype effects.	Liu et al., 2019 ^[^ ^59^ ^]^
DeepHF (RNN)	RNN	Predict gRNA on‐target efficiency	Data scarcity makes it difficult to assess which algorithms perform best in specific biological contexts.	Wang et al., 2019 ^[^ ^68^ ^]^
Cas13 Guide RNA Design	RF	On‐target efficiency	Optimized for CDS, less effective in noncoding regions, lacks RNA structure and spliceosome effects.	Wessels et al., 2020 ^[^ ^69^ ^]^
CnnCrispr	CNN and biLSTM	sgRNA off‐target propensity prediction	CnnCrispr's limitations include data dependency, limited generalizability across genomic contexts, computational complexity, and challenges in predicting off‐target effects or ensuring interpretability.	Liu et al., 2020 ^[^ ^70^ ^]^
DL‐CRISPR	CNN	Predict off‐target activity in CRISPR/Cas9 gene editing.	DL‐CRISPR's limitations include reliance on large datasets, limited generalizability, high computational cost, and challenges in predicting off‐target effects and ensuring interpretability.	Zhang et al., 2020 ^[^ ^71^ ^]^
CNN‐SVR (hybrid)	CNN and SVR	CRISPR/Cas9 guide RNA (gRNA) on‐target cleavage efficacy prediction	High computational complexity, dependency on large labeled datasets, limited interpretability, and potential overfitting from hybrid architecture tuning challenges.	Zhang et al., 2020 ^[^ ^72^ ^]^
BE‐Hive	GBRT, Deep autoregressive model	On‐target efficiencyˆ, Editing outcome	Limitation is reduced accuracy in predicting rare or complex editing events and potential dependency on cell‐type‐specific experimental conditions.	Arbab et al., 2020 ^[^ ^61^ ^]^
DeepPE	CNN	On‐target efficiency	DeepPE struggles to generalize well beyond its training dataset, leading to lower performance on external datasets​.	Kim et al., 2021 ^[^ ^73^ ^]^
PE_type, PE_Position	RF	On‐target efficiency	PE_type and PE_Position models are biased by position‐dependent sequence preferences and varying editing efficiencies, causing inaccuracies in predicting editing outcomes.	Kim et al., 2021 ^[^ ^73^ ^]^
CROTON	CNN, NAS	On‐target efficiency, Editing outcome	Only predicts Deletion frequency,1 bp Insertion/Deletion, 1/2 bp Frameshift frequency, Frameshift frequency.	Li et al., 2021 ^[^ ^74^ ^]^
Apindel	GloVe, BiLSTM (RNN), Attention	On‐target efficiency, Editing outcom	Only predicts 536 classes of Deletions, 21 classes of Insertions	Liu et al., 2022 ^[^ ^75^ ^]^
FORECasT‐BE	GBRT	On‐target efficiency	FORECasT‐BE struggles with unintended edits, particularly at non‐targeted cytosines and adenines, which pose challenges for clinical applications​	Pallaseni et al., 2022 ^[^ ^76^ ^]^
CRISPR‐OTE	CNN and biLSTM	gRNA on‐target efficiency prediction	Requires high‐quality off‐target labels; less effective for low‐frequency off‐targets.	Xie et al., 2023 ^[^ ^77^ ^]^
CrisprDNT	CNN, LSTM, and transformer	off‐target activity prediction	Limited generalization and potential data bias affect scalability and noise robustness	Guan and Jiang, 2023 ^[^ ^78^ ^]^
ABEdeepoff and CBEdeepoff	biLSTM	Prediction of base editor off‐targets	Library design excludes sequences without editable nucleotides, limiting predictions for such cases.	Cheng dong et al., 2023 ^[^ ^79^ ^]^
PREDICT	Attention‐based bidirectional RNN	On‐target efficiency, Editing outcome	Performance may drop under different experimental conditions or chromatin contexts.	Mathis et al., 2023 ^[^ ^80^ ^]^
OpenCRISPR‐1	Large Language Models (LLMs)	Accurate on target prediction	Performance may vary across experimental conditions and delivery methods.	Jeffrey et al., 2024 ^[^ ^49^ ^]^
DeepCRISTL	LSTM	predict CRISPR/Cas9 on‐target editing efficiency	DeepCRISTL's drawbacks include limited generalization, reliance on low‐correlation data, high computational costs, and underuse of biological features.	Elkayam et al., 2024 ^[^ ^81^ ^]^
CrnnCrispr	CNN	It predicts sgRNA on‐target activity for efficient and safe genome editing.	Relies solely on sgRNA sequences, omitting cell‐type‐specific biological features (e.g., DNA methylation, chromatin accessibility).	Zhu et al., 2024 ^[^ ^82^ ^]^
CRISPR‐M	CNN Bi‐LSTM	Predicting sgRNA off‐target effect	The model has limited predictive accuracy under extreme epigenetic modifications and requires further validation for generalizability across different cell types.	Sun et al., 2024 ^[^ ^83^ ^]^
CRISPR‐DIPOFF	LSTM	Predicting CRISPR Cas‐9 off‐target	The model does not consider indel mismatches and has only been trained on a single dataset, potentially limiting its generalizability​	Toufikuzzaman et al., 2024 ^[^ ^84^ ^]^
CrisprBERT	BERT BiLSTM	CrisprBERT predicts off‐target effects of CRISPR‐Cas9 sgRNAs, improving genome editing specificity.	CrisprBERT shows only modest performance improvement and requires more diverse datasets to better generalize across different experimental conditions.	Sari et al., 2024 ^[^ ^60^ ^]^
CRISPR‐BERT	BERT CNN BiGRU	CRISPR‐BERT predicts off‐target activities, including mismatches and indels, to enhance specificity.	CRISPR‐BERT needs large, diverse data for better generalization and is computationally intensive.	Luo et al., 2024 ^[^ ^85^ ^]^
Crispr‐SGRU	CNN BiGRU	Crispr‐SGRU predicts CRISPR‐Cas9 off‐target activities, including mismatches and indels, to improve accuracy.	Crispr‐SGRU requires more diverse and experimentally validated datasets for better generalization and remains computationally intensive due to deep learning complexity.	Zhang et al., 2024 ^[^ ^86^ ^]^
CRISPR‐MCA	CNN	Used for predicting CRISPR‐Cas9 off‐target effects to enhance gene‐editing accuracy and safety.	Although CRISPR‐MCA performs well across datasets, it is still limited by dataset imbalance and the complexity of off‐target effect prediction.​	Yang et al., 2024 ^[^ ^87^ ^]^
DeepMEns	CNN, Transformer, LSTM, Attention Mechanism	Predicts CRISPR‐Cas9 sgRNA on‐target activity to improve gene‐editing efficiency and precision.	DeepMEns performs well across datasets but is limited by data heterogeneity and experimental variations.​	Ding et al., 2025 ^[^ ^88^ ^]^
sgRNA design				
DeepCRISPR	CNN	Predict on‐ and off‐target efficiency	The model's understanding of important features for sgRNA structure can be limited when trained with a small number of samples.	Chuai et al., 2018 ^[^ ^57^ ^]^
DeepCas9	CNN	Identifying CRISPR‐Cas9 sgRNA activity directly from genetic sequences	Focuses on clinical assays; real‐world application may vary across different experimental settings.	Xue et al., 2018 ^[^ ^89^ ^]^
DeepSgRNA	CNN	To predict the efficiency of sgRNAs in the CRISPR‐Cas9 system	The study does not take into account off‐target effects for specific sgRNAs, limiting its comprehensive application.	Shrawgi et al., 2019 ^[^ ^90^ ^]^
CNN with 5layers +transfer learning	CNN	Predicting sgRNA activity in prokaryotic and eukaryotic species.	Model does not perform well for predicting on‐target activity in some specific Cas9 variants.	Wang et al., 2019 ^[^ ^91^ ^]^
AttCRISPR	CNN &RNN	To predict sgRNA on‐target activity	Relies on high‐quality labeled data, struggles with generalizability to novel genomic contexts, computationally intensive, and partial interpretability despite attention layers.	Xiao et al., 2021 ^[^ ^92^ ^]^
TransCrispr	Transformer and CNN	Predicting CRISPR/Cas9 Single Guide RNA Cleavage Efficiency	Computationally heavy; limited interpretability of transformer‐based predictions.	Wan et al., 2023 ^[^ ^63^ ^]^
EXPosition	CNN LSTM SVM	Enhancing CRISPR sgRNA knockout prediction by integrating gene expression models with existing tools.	Potential bias toward coding regions and high computational demands for large‐scale data.	Cohen et al., 2024 ^[^ ^93^ ^]^
Predict editing efficiency				
BoostMEC	LightGBM	predicting CRISPR‐Cas9 cleavage efficiency	BoostMEC's limitations include limited validation to wild‐type CRISPR‐Cas9 with U6/T7 promoters and no testing on other systems, promoters, or non‐animal cells.	Zarate et al., 2022 ^[^ ^94^ ^]^
DeepCas9variants	‐	predict the editing efficiencies and outcomes of diverse base editors	The model, while effective in predicting Cas9 variant efficiency, is still constrained by the quality and diversity of training data, which may limit its applicability to unseen target sequences.	Kim et al., 2024 ^[^ ^95^ ^]^
BE_Seq BE_Endo	CNN	It predicts the editing efficiency of ABE and CBE at genomic target sites to improve base editing accuracy.	The model's accuracy is affected by endogenous factors, and its generalization to diverse contexts needs further validation.	Yuan et al., 2024 ^[^ ^96^ ^]^
DeepIndel	BERT‐based deep learning model Deep SHAP	Predicting CRISPR/Cas9‐Mediated Editing Outcomes	The model shows limited performance in predicting repair outcomes under extreme chromatin modifications and struggles to generalize across different cell types.	Zhang et al., 2024 ^[^ ^62^ ^]^
BEguider	CNN Bi‐LSTM	The model predicts editing efficiency and outcomes of near‐PAMless base editors, optimizing their design for disease research and clinical use.​	The model underperforms at sites with consecutive A or C and has limited generalizability across non‐HEK293T cell lines.	Zhou et al., 2024 ^[^ ^97^ ^]^
igRNA‐PS	CNN Transfer Learning	This model predicts and optimizes the editing efficiency and specificity of near‐PAMless ABEs, reducing bystander editing and improving precision.	The model has limited generalizability when predicting editing efficiency in non‐HeLa cell lines, and its performance in specific editing windows requires further optimization​.	Li et al., 2024 ^[^ ^98^ ^]^
panCRISPR Toolbox	GBR, and SVM	improve CRISPR/Cas experiments	Limited cross‐species generalization, low BLAST efficiency, and lack of a user‐friendly interface and visualization tools.	Yasafova et al., 2022 ^[^ ^99^ ^]^
TEEP	CNN, RNN	Predicting ωRNA activity of ISDra2 TnpB to optimize genome‐editing designs	Relies on experimental data scale and may not fully account for in vivo complexities.	Marquart et al., 2024 ^[^ ^100^ ^]^
predicted the activity of the Cas9 and//or CRISPR				
DeepSpCas9	DNN CNN	It effectively predicted the activity of the SpCas9 enzyme with high accuracy	The training dataset used was insufficient, affecting the model's overall performance.	Kim et al., 2019 ^[^ ^101^ ^]^
DeepAcr	CNN &DNN	Predicting Anti‐CRISPR	DeepAcr's shortcomings include a small dataset, high AlphaFold costs, challenges in 3D structure representation, and poor interpretability.	Li et al., 2022 ^[^ ^102^ ^]^
AcrTransAct	transformer‐based deep neural network	predict Acr‐mediated CRISPR‐Cas inhibition	limited by its focus on Type I CRISPR‐Cas systems, a small dataset, and potential overfitting in larger models.	Hasani et al., 2023 ^[^ ^103^ ^]^

#### 3.5.1 AI in Predicting CRISPR On‐Target Efficiency

AI has demonstrated exceptional performance in predicting the on‐target efficiency of CRISPR systems, which determines the ability of genome editing tools to cut or modify DNA at the intended location. One of the earliest and most significant models in this field is DeepCRISPR,^[^ ^57^ ^]^ which uses convolutional neural networks (CNNs) to simultaneously predict both on‐target and off‐target efficiency of the CRISPR‐Cas9 system. A key innovation of DeepCRISPR was its attempt to balance editing efficiency and safety within a single model, addressing the challenge where higher targeting efficiency often comes at the cost of increased off‐target risks. However, its performance was limited when training data was insufficient, highlighting AI's strong dependence on high‐quality, large‐scale datasets.

A contemporary model, DeepCas9, also based on CNNs, focused on directly identifying gRNA activity from genetic sequences. While it showed promising applications in clinical diagnostics, variability across different experimental conditions revealed the challenge of AI models in generalizing across diverse biological contexts.

#### 3.5.2 AI in Reducing Off‐Target Effects

AI has also played a key role in enhancing the specificity of genome editing by reducing off‐target effects, which could lead to unintended DNA modifications and safety concerns. In 2019, AttnToMismatch_CNN introduced a breakthrough approach by combining CNNs with attention mechanisms to predict gRNA specificity.^[^ ^59^ ^]^ The attention mechanism enabled the model to focus on sequence regions most relevant to off‐target effects, improving prediction accuracy. However, challenges such as high data dependency, limited generalizability, and computational complexity remain, especially in more intricate AI models.

By 2024, CrisprBERT,^[^ ^60^ ^]^ a model combining BERT (a Transformer‐based model) with a BiLSTM (bidirectional long short‐term memory network), further improved off‐target prediction accuracy. While the model showed incremental improvements, its performance remained constrained by the need for more diverse datasets to enhance cross‐species and cross‐cell‐line generalizability. Nonetheless, CrisprBERT represents a frontier exploration of AI in CRISPR specificity control.

#### 3.5.3 AI in Predicting Editing Outcomes

AI has also achieved significant advancements in predicting genome editing outcomes, particularly in forecasting insertions or deletions (indels) and base modifications following CRISPR editing. A notable model, BE‐Hive,^[^ ^61^ ^]^ integrates gradient boosting regression trees (GBRT) and deep autoregressive models to predict the efficiency and outcomes of base editing. While BE‐Hive performed well in predicting common editing events, its accuracy for rare or complex editing events remained low, and it was heavily dependent on cell‐type‐specific data, underscoring the challenge of accurate genetic modification predictions.

In 2024, DeepIndel,^[^ ^62^ ^]^ built on a BERT‐based deep learning architecture, demonstrated impressive performance in predicting CRISPR‐Cas9‐mediated repair outcomes. However, its stability varied significantly under extreme chromatin modifications and across different cell types, again highlighting the limitations of AI models in adapting to diverse biological environments.

#### 3.5.4 AI in gRNA Design and Next‐Generation Genome Editing

AI is also driving innovation in gRNA design and next‐generation genome editing technologies. TransCrispr,^[^ ^63^ ^]^ a model combining Transformers with CNNs, optimized Cas9 gRNA cutting efficiency. While it introduced more complex architectures, it also posed higher computational demands and limited interpretability. OpenCRISPR‐1^[^ ^49^ ^]^ leveraged large language models (LLMs) to achieve high‐precision targeting predictions. LLMs excel at processing vast datasets and capturing intricate sequence patterns, yet performance variability across experimental conditions suggests further optimization is needed.

#### 3.5.5 AI's Expanding Role in Genome Editing

Overall, AI‐driven approaches, powered by CNNs, Transformers, and LLMs, have been integrated into multiple aspects of genome editing—from on‐target efficiency prediction and off‐target control to editing outcome optimization—demonstrating tremendous application potential. Despite challenges such as data dependency, generalization limitations, and computational complexity, AI has significantly improved CRISPR tool performance, unlocking new possibilities for precision medicine, agricultural biotechnology, and synthetic biology. As datasets expand and AI algorithms continue to evolve, AI is poised to drive genome editing toward higher efficiency and precision, shaping the future of genetic engineering with profound impacts on healthcare and agriculture.

## 4 The Advantages of AI‐powered Genome Editing

Compared to traditional genome editing algorithms, AI‐powered genome editing demonstrates significant advantages across four key aspects: 1) Enabling efficient discovery of novel editing tools. Traditional approaches for discovering novel genome editing components have largely relied on sequence homology searches and manually intensive experimental validation. In contrast, AI‐enabled methods using advanced protein language models (e.g. AlphaFold2,^[^ ^5^ ^]^ ProtT5,^[^ ^104^ ^]^ ESM^[^ ^105^ ^]^), are capable of analyzing vast protein sequence and structural datasets. These models can identify subtle patterns and predict structural functional relationships that would be difficult to discern with conventional computational tools. This capability not only accelerates the discovery of novel Cas and deaminase enzymes but also improves the identification of potential effector proteins by screening for functional motifs across diverse species. For example, Feng Zhang's team developed the novel RNA‐guided system TIGR‐Tas^[^ ^106^ ^]^ using AI models, while Caixia Gao's team leveraged the AlphaFold2 model to discover new deaminases.^[^ ^56^ ^]^ 2) Editing outcome prediction. Genomic data exhibits high‐dimensional and nonlinear characteristics. Traditional genome‐editing algorithms based on statistical or linear models struggle to comprehensively analyze the high‐dimensional nonlinear features of genomic data, resulting in constrained predictive performance. AI models (such as the gradient‐boosted tree‐based Elevation‐score^[^ ^107^ ^]^) can more effectively capture the complex interactions between genomic features and cellular environments, achieving ≈7% improvement in AUC metrics and outperforming traditional methods (e.g., CCTOP^[^ ^108^ ^]^). These AI approaches demonstrate superior accuracy and generalization capability in off‐target risk prediction. 3) Designing gRNA to be smarter and more efficient. Conventional methods for guide RNA (gRNA) design tend to rely on sequence alignment algorithms and simple feature‐based scoring. DeepCRISPR^[^ ^57^ ^]^ integrates convolutional neural networks (CNNs) to learn complex sequence‐activity relationships from large‐scale experimental data, enabling the capture of nonlinear features that are challenging for traditional methods to model. This approach enhances the specificity and effectiveness of gRNA design. Compared to conventional gRNA design tools based on sequence alignment, thermodynamic models, and genomic annotation, such as E‐CRISP^[^ ^109^ ^]^ and CHOPCHOP,^[^ ^110^ ^]^ DeepCRISPR^[^ ^57^ ^]^ achieves over 18% improvement in On‐Target ROC‐AUC values. This demonstrates that AI models can more precisely predict optimal gRNAs and significantly increase editing success rates. 4) Superior capability in multi‐omics integration and predictive modeling. AI possesses inherent advantages in processing large‐scale, high‐dimensional, and multi‐omics datasets. In particular, generative models such as Transformers demonstrate strong adaptability and integration capabilities, enabling the effective fusion of epigenomic, transcriptomic, and proteomic information to uncover regulatory networks and key genome editing targets tasks that traditional linear regression models and homology‐based tools struggle to accomplish. For example, the EMOGI^[^ ^50^ ^]^ algorithm successfully identified novel cancer gene targets through multi‐omics data integration, a process that would require significantly more time and experimental resources using conventional approaches.

## 5 Ethical Considerations of AI and Genome Editing

**Figure 6:**
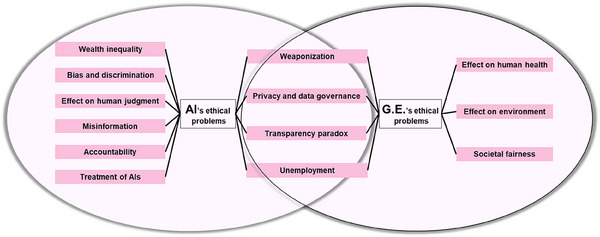
Ethical concerns regarding AI and genome editing.

The integration of AI into genome editing technologies has amplified both the technical capabilities and ethical complexities of this field.^[^ ^111^ ^]^ While AI‐driven tools like AlphaFold^[^ ^112^ ^]^ and DeepCRISPR^[^ ^57^ ^]^ enhance the precision of CRISPR‐Cas9 systems by predicting off‐target effects and optimizing guide RNA designs, they also raise novel ethical concerns. A primary issue involves the dual‐use dilemma: AI‐accelerated genome editing could be exploited for non‐therapeutic purposes, such as human enhancement or biological weapon development, and it may exacerbate social or income disparities, leading to inequities (**Figure** **6**). Another critical concern is algorithmic bias in AI tools, which may exacerbate healthcare disparities. The training datasets for AI‐based CRISPR design often underrepresent all ethnic groups, leading to unequal efficacy and safety outcomes across ethnic groups. Furthermore, the opaque “black‐box” nature of many AI systems complicates accountability. The lack of transparency in AI‐driven editing platforms challenges informed consent processes, as patients and regulators cannot fully assess risks tied to algorithmic decisions. Currently, informed consent is widely recognized as a key foundation of research on humans and is a major tool in bioethics for the protection of human rights and human dignity. In the future, AI technology should be leveraged to enhance informed consent agreements.

These challenges underscore the urgent need for robust ethical guidelines and governance frameworks specifically tailored to AI‐enhanced genome editing. While some existing bioethical principles and international declarations offer a foundational basis, they must be further updated and integrated to address the unique dilemmas introduced by AI. As AI continues to redefine the possibilities of genome editing, a coordinated global approach balancing innovation, equity, and accountability will be essential to ensure that technological progress serves the collective good of humanity.

## 6 Opportunities and Future Challenges

The rise of protein language models (e.g., ESM,^[^ ^105^ ^]^ ProtT5,^[^ ^104^ ^]^ ProtBert,^[^ ^104^ ^]^ ProtTXL,^[^ ^104^ ^]^ ProtAlbert,^[^ ^104^ ^]^ ProGen^[^ ^113^ ^]^) and DNA language models (e.g., DNABERT,^[^ ^114^ ^]^ Nucleotide Transformers,^[^ ^115^ ^]^ HyenaDNA,^[^ ^116^ ^]^ PDLLMs,^[^ ^117^ ^]^ AgroNT,^[^ ^118^ ^]^ Evo2^[^ ^119^ ^]^) has injected new vitality into genome editing, providing innovative ideas and novel methodologies for the improvement and optimization of genome‐editing tools. For instance, leveraging protein language models such as ProtT5 and ESM to analyze amino acid sequences of Cas proteins and deaminases, extract embeddings, and integrate multimodal information (e.g., enrichment analysis and protein regulatory networks), researchers can combine these approaches with natural language processing models like Transformer and BERT to develop intelligent algorithms for Cas protein and deaminase recognition. These algorithms enable precise identification of critical functional sites, systematic mining of novel Cas enzymes and deaminases from vast protein datasets, and screening of high‐specificity variants. Such advancements facilitate both the optimization of existing genome‐editing tools and the development of next‐generation editing systems.

Genome editing requires high computational power. For example, one research optimizes the Smith–Waterman algorithm using GPU and hybrid CPU‐GPU computing to significantly accelerate CRISPR‐related DNA sequence analysis tasks. Experimental results show that GPU computing can drastically reduce computation time. For large‐scale DNA sequence analysis, GPU computing is 18 times faster than sequential CPU execution, while hybrid CPU‐GPU computing further improves performance to 24 times, making large‐scale genome alignment more feasible.^[^ ^120^ ^]^ In the future, it is recommended to integrate multimodal models by jointly training DNA language models (e.g., DNABERT) with protein models, while incorporating architectures like Graph Neural Networks (GNNs) and Transformers. This approach could enable simultaneous optimization of sgRNA and Cas protein design, facilitating the development of novel gene‐editing tools with enhanced editing efficiency. Large language models, with billions or even hundreds of billions of parameters, require substantial computational resources for training and inference, posing a significant challenge in terms of computational load. It is recommended to address this challenge through methods such as model compression, algorithm optimization, distributed computing, and hardware acceleration. Additionally, it is recommended that biologists collaborate with technology companies such as Google and Tencent, which can provide the computational resources required for model training, thereby accelerating the development of algorithms.

In some applications, AI model predictions lack sufficient reproducibility. For example, when validated using independent datasets from different laboratories, the prediction accuracy of the DeepCRISPR tool may decrease by over 30%. Specifically, the model trained on HCT116 colon cancer cell data showed a drop in AUC value from 0.92 to 0.68 when applied to human iPSC (induced pluripotent stem cell) data from another research team. These discrepancies primarily arise from differences in sgRNA libraries and quantification methods for editing efficiency adopted across laboratories. We suggest strengthening standardized experimental designs and cross‐research team data sharing to ensure the reliability of AI in the field of genome editing. Currently, Cas protein and its variants, gRNA, and target gene datasets exhibit biases or limitations, such as the predominance of human and animal genome editing data compared to plant‐related datasets. To address this, on the one hand, deciphering high‐quality Telomere‐to‐Telomere (T2T) genomes enables the resolution of genetic information in gap regions and allows for more comprehensive mining of target genes. On the other hand, it is recommended to introduce algorithms like Generative Adversarial Networks (GANs) to generate synthetic data, thereby enhancing dataset diversity. Additionally, leveraging models such as ProtBERT and ProtT5^[^ ^104^ ^]^ for cross‐species learning and fine‐tuning on data from different organisms could improve model performance in non‐human species and broaden their applicability. Transfer learning would particularly aid CRISPR design in species beyond humans by enabling models to adapt to distinct genomic features. Looking ahead, integrating multi‐omics information, including transcriptomic, proteomic, and genomic data, should be prioritized to comprehensively assess the biological impacts of genetic variations. Such integration would empower AI models to better support gene‐editing decisions, especially in personalized medicine and disease research.

Additionally, large‐scale genetic engineering pipelines often involve extensive gene editing experiments. AI algorithms, such as Transformer models, can automate the screening and analysis of gene targets, optimize CRISPR designs, and identify optimal editing strategies, thereby saving experimental time and costs. In the future, AI algorithms could be employed to construct real‐time learning systems. For instance, by leveraging architectures like AutoGPT, researchers might develop CRISPR design assistants capable of autonomously updating their knowledge bases. Through real‐time learning and optimization, AI can play a pivotal guiding role in large‐scale genetic engineering workflows. Using artificial intelligence (AI) technology to develop predictive algorithms can enable precise prediction of genome editing outcomes. For example, the DeepCRISPR tool, developed based on the CNN algorithm, can predict the targets and off‐target effects of different gRNAs, thereby forecasting the effects of genome editing. The DTMP‐Prime^[^ ^40^ ^]^ model, developed using DNABERT and Transformer algorithms, guides PegRNA design to ensure efficient editing while avoiding unnecessary off‐target effects. In the future, it is recommended to integrate multimodal information, such as DNA sequences, RNA structures, and protein structures, combining DNA language models and protein language models for cross‐domain data integration and analysis. Using GNN and Transformer algorithms, an automated genome editing guidance model can be developed to accurately predict editing outcomes, quickly screen the most effective editing tools and strategies, and improve editing efficiency.

The AI black‐box problem in genome editing is both a technical bottleneck and an opportunity to drive interdisciplinary innovation. To improve model transparency, efforts should be made to open‐source the models. To improve model interpretability, it is recommended to incorporate biological background knowledge into model design to help AI models understand the biological relevance of genome editing tasks. For example, known genomic functional regions, regulatory elements, and other biological information can be integrated to guide the learning process of the model, thereby enhancing the biological significance of its predictions.

## 7 Concluding Remarks

The emergence of AI has significantly influenced various research fields, particularly those dealing with vast datasets like functional genomics. The application potential of AI in GEd are remarkably promising, offering insights into genome functionality and regulatory mechanisms, thereby enhancing support and guidance for precision medicine, agricultural and environmental conservation. However, the current research in this field is primarily nascent, showcasing emerging trends of study, necessitating scholars' continued attention and dedication to further exploration. Moreover, research endeavors in this area demand collaborative efforts among scholars specializing in bioinformatics, biotechnology, and biochemistry, which present inherent complexities and challenges. Nevertheless, the optimism for progress and advancements in this field remains substantial.

## Conflict of Interest

The authors declare no conflict of interest.

## Author Contributions

F.C. and M.L. designed this research. Z.L. and F.C. performed the analyses and wrote the draft MS. W.U.K., G.B., C.D., H.Z., J.W., Y.Z., C.W., and W.W. participated in the paper reviewing and drafting. All the authors read and approved the final MS.
